# CRISPR/Cas9‐mediated mutagenesis to validate the synergy between PARP1 inhibition and chemotherapy in *BRCA1*‐mutated breast cancer cells

**DOI:** 10.1002/btm2.10152

**Published:** 2020-01-02

**Authors:** Rachel L. Mintz, Yeh‐Hsing Lao, Chun‐Wei Chi, Siyu He, Mingqiang Li, Chai Hoon Quek, Dan Shao, Boyuan Chen, Jing Han, Sihong Wang, Kam W. Leong

**Affiliations:** ^1^ Department of Biomedical Engineering Columbia University New York New York; ^2^ Department of Biomedical Engineering CUNY‐City College of New York New York New York; ^3^ Laboratory of Biomaterials and Translational Medicine The Third Affiliated Hospital, Sun Yat‐sen University Guangzhou China; ^4^ State Key Laboratory of Microbial Resources Institute of Microbiology, Chinese Academy of Sciences Beijing China; ^5^ Department of Systems Biology Columbia University Medical Center New York New York

**Keywords:** BRCA1, CRISPR/Cas9, PARP1, precision medicine, synthetic lethality, triple negative breast cancer

## Abstract

For patients carrying *BRCA1* mutations, at least one‐third develop triple negative breast cancer (TNBC). Not only is TNBC difficult to treat due to the lack of molecular target receptors, but *BRCA1* mutations (BRCA1m) also result in chemotherapeutic resistance, making disease recurrence more likely. Although BRCA1m are highly heterogeneous and therefore difficult to target, *BRCA1* gene's synthetic lethal pair, *PARP1*, is conserved in BRCA1m cancer cells. Therefore, we hypothesize that targeting *PARP1* might be a fruitful direction to sensitize BRCA1m cancer cells to chemotherapy. We used CRISPR/Cas9 technology to generate *PARP1* deficiency in two TNBC cell lines, MDA‐MB‐231 (*BRCA1* wild‐type) and MDA‐MB‐436 (BRCA1m). We explored whether this *PARP1* disruption (PARP1m) could significantly lower the chemotherapeutic dose necessary to achieve therapeutic efficacy in both a 2D and 3D tumor‐on‐a‐chip model. With both BRCA1m and PARP1m, the TNBC cells were more sensitive to three representative chemotherapeutic breast cancer drugs, doxorubicin, gemcitabine and docetaxel, compared with the *PARP1* wild‐type counterpart in the 2D culture environment. However, PARP1m did not result in this synergy in the 3D tumor‐on‐a‐chip model, suggesting that drug dosing in the tumor microenvironment may influence the synergy. Taken together, our results highlight a discrepancy in the efficacy of the combination of PARP1 inhibition and chemotherapy for TNBC treatment, which should be clarified to justify further clinical testing.

1

Breast cancer is the leading cause of death in women worldwide.^1^ Around 12–17% of breast cancer patients have triple negative breast cancer (TNBC),^2^ an aggressive, heterogeneous subtype characterized by the lack of estrogen receptor (ER), progesterone receptor (PR) and human epidermal growth factor receptor Type 2 (HER2) expression. Due to TNBC heterogeneity and lack of specific markers for targeted endocrine therapy, chemotherapy is usually the only feasible treatment option.^3^ The therapeutic outcome is limited, and TNBC tumors often develop resistance. Consequently, TNBC results in the poorest overall survival of any other breast cancer subtype.^4^ To achieve more successful prognoses, there is a clinical need to develop more tailored treatments against TNBC.

Overall, 5–10% of breast cancers are attributed to the inheritance of a mutation in the tumor suppressor *BRCA1* gene (BRCA1m).^5^ Yet, up to 70–90% of BRCA1m carriers develop TNBC.^6^ There are variable forms of BRCA1m, which increases the difficulty of potentially targeting those specific mutations for TNBC therapy. The poly (ADP‐ribose) polymerase 1 (PARP1) gene, the synthetic lethal pair of *BRCA1*, however, is conserved in most of the BRCA1m cancer cells and thus may be a fruitful target for TNBC therapy.^7^ Neither PARP1 inhibition alone nor *BRCA1* deficiency alone is lethal, but the combination of the two is, suggesting a therapeutic strategy that leverages this synthetic lethality.

PARP enzymes are mainly involved in single‐stranded DNA break repair, while BRCA1 plays a role in several pathways of DNA repair, including homologous recombination repair (HR) and nonhomologous end joining repair (NHEJ) of double‐stranded DNA breaks. PARP1 inhibition results in the accumulation of single‐stranded DNA breaks, which leads to the stalling of replication forks. Since repair mechanisms are not present in BRCA1m cells, these stalled replication forks degrade, forming double‐stranded DNA breaks.^8^ Typically, the double‐stranded DNA breaks would be repaired through either the HR or NHEJ pathway. However, BRCA1m and PARP1 inhibition cause HR initiation failure. The error‐prone NHEJ repair pathway predominates, culminating in genomic instability, and ultimately cell death.^7^

As a potential approach for treating TNBC with BRCA1m, several PARP1 inhibitors, such as olaparib (AZD‐2281), and veliparib (ABT‐888), are under investigation in clinical trials. Olaparib has demonstrated clinical efficacy,^9^ earning approval for the treatment of germline BRCA1m, metastatic breast cancer. Nevertheless, PARP1 inhibitor monotherapy has shown mixed success in clinical trials. In a 2011 phase II clinical trial (NCT00679783), for example, olaparib monotherapy did not improve the response rate in TNBC patients, including patients with a germline *BRCA1* or *BRCA2* mutation.^10^

Studies of PARP1 inhibition in conjunction with chemotherapy have consequently been tested in clinical trials as a means to improve the therapeutic efficacy, but similarly, limited improvement was found.^11^ The combinational therapeutic success may be mediated by several variables including: the type of PARP1 inhibitor, the pharmacokinetic properties of the combinational chemotherapeutic drugs, the suboptimal dosage, and the patients' genetic profiles. While the genetic synthetic lethality paradigm may hold therapeutic promise for TNBC, combining PARP1 inhibitor drugs with chemotherapy to take advantage of this genetic relationship may be more challenging than anticipated.

Given the inconsistent clinical data, CRISPR technology may be an expedient tool to confirm drug specificity in preclinical studies prior to clinical testing. Particularly, compared with other gene manipulation strategies, such as antagonists or RNAi, CRISPR‐mediated gene manipulation is more precise^12^ and may have comparably fewer off‐targeting effects.^13^ Recently, despite ongoing clinical trials using Maternal Embryonic Leucine Zipper Kinase (MELK) inhibitors as chemotherapeutics, a study used CRISPR technology to disrupt MELK in vitro, debunking the notion that MELK was necessary for basal breast cancer cell fitness.^14^ By undermining the rationale for current clinical trials, this study corroborates the need for using CRISPR technology in preclinical target validation. Inspired by the aforementioned study, we optimize the CRISPR/Cas9 system to target the *PARP1* gene for validation of the selective synergism between *PARP1* disruption and chemotherapy in TNBC cells. We tested different *BRCA1* and *PARP1* genetic profiles in an in vitro 2D setting as well as in a 3D tumor‐on‐a‐chip system^15^ to better mimic a physiological setting (Scheme 1).

**Scheme 1:**
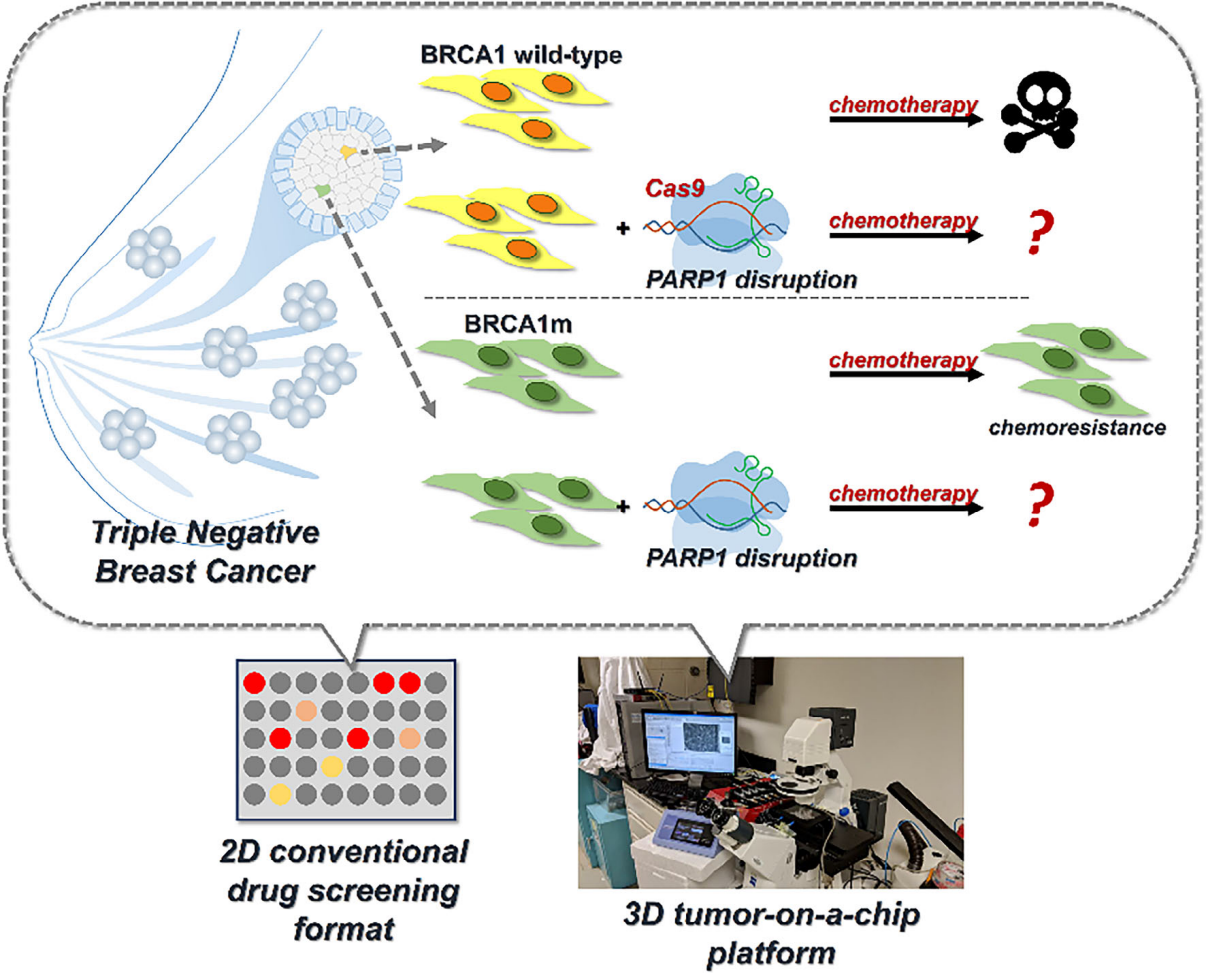
Hypothesis and design of this study that applies CRISPR/Cas9‐mediated *PARP1* mutagenesis for validating the *BRCA1* wild‐type and BRCA1m TNBC cellular response to *PARP1* disruption and chemotherapy

We first tested the response of the *BRCA1* wild‐type (WT) TNBC cell line, MDA‐MB‐231, and the BRCA1m line, MDA‐MB‐436 (containing a c.5396 + 1G > A mutation^16^) against two PARP1 inhibitors, olaparib and veliparib. In 2D culture, MDA‐MB‐436 was only slightly more sensitive to both PARP1 inhibitors than MDA‐MB‐231, while in 3D, the difference in senstivity to veliparib between the cell lines was even smaller (Figure S1). These results are in accordance with the findings from a previous study that also only showed a minor difference in the drug IC50 dose for these cell lines.^17^ Because these are two cancer cell lines, they may have variable additional chromosomal mutations, culminating in genetic differences between the two TNBC cell lines. These genetic inconsistencies between the two cells lines may explain differences in sensitivity to PARP1 inhibitors. Thus, comparing the effects of PARP1 inhibitors on two heterogeneous cell lines (MDA‐MB‐436 and MDA‐MB‐231) may not be valid. This realization further justifies the use of CRISPR technology for this study, which can introduce a single gene disruption (*PARP1*) to generate a modified cell line. This singly mutated cell line can then be evaluated with the nonmodified, otherwise identical, cell line from which it is derived for valid, pairwise comparisons (MDA‐MB‐436 vs. MDA‐MB‐436‐*PARP1*‐mutated and MDA‐MB‐231 vs. MDA‐MB‐231‐*PARP1‐*mutated).

To validate this genetic paradigm, we then designed the CRISPR/Cas9 system to disrupt the *PARP1* gene in both cell lines. The guide RNAs (gRNAs) targeting *PARP1* (Figure 1a) were selected using the CHOPCHOP algorithm in the default setting.^18^ Based on the predicted efficiency and off‐targeting effects, the top three resultant gRNA candidates (see Table S1 for the sequences) were synthesized by in vitro transcription with an optimized gRNA backbone^19^ and then transfected with Cas9 plasmid in HEK cells for gene disruption evaluation. The result of the T7 endonuclease I (T7EI) assay indicated that gRNA1 was the most efficient among the three candidates; the *PARP1* disruption efficiencies with gRNA2 and gRNA3 only reached 82 and 23% of that with gRNA1, respectively (Figure 1b). The mutation on exon 7 caused by gRNA1 may lead to a frameshift on the domain C of the PARP1 enzyme, disrupting its DNA‐binding capability and enzymatic activity.^20^ The gRNA1 was subsequently cloned into an all‐in‐one Cas9‐T2A‐EGFP plasmid^21^ using our previously established protocol^22^ for *PARP1* mutated (PARP1m) TNBC cell generation (sequence verified by Sanger sequencing, Figure S2).

**Figure 1:**
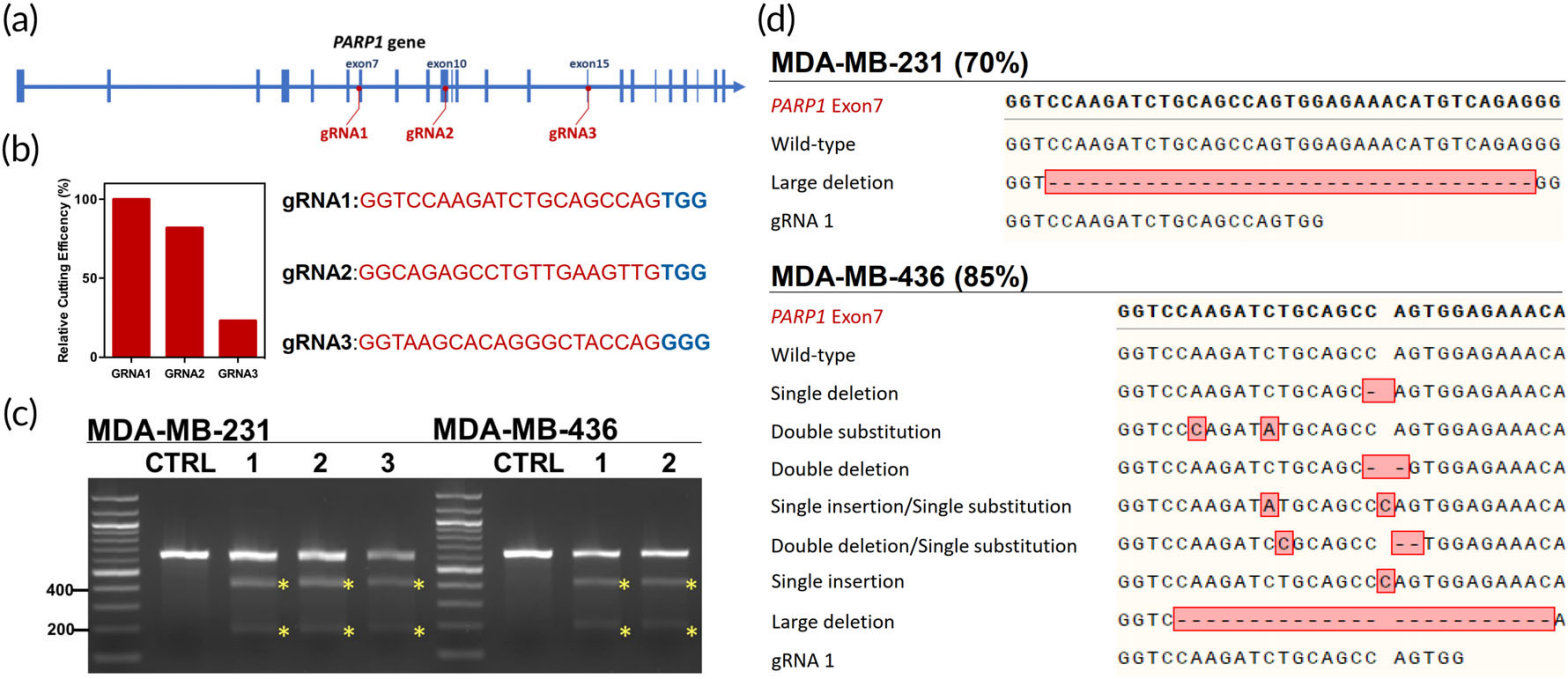
PARP1m breast cancer cell line generation. (a) Exon targets of the gRNA used in this study. (b) Relative *PARP1* gene disruption efficiency of the gRNA candidates. (c) T7EI validation of *PARP1* gene disruption after multiple selections in MDA‐MB‐231 and MDA‐MB‐436 cell lines. (d) Sanger sequencing validation of *PARP1* gene disruption post selections

We first generated the PARP1m, *BRCA1* WT MDA‐MB‐231 cell line by transfecting the gRNA1‐encoding Cas9‐T2A‐EGFP plasmid and collecting the live GFP^+^ cells by cell sorting. Multiple selections were subsequently conducted to enrich the PARP1m population. As shown in Figure 1c, CRISPR/Cas9‐mediated *PARP1* mutagenesis was detected by T7EI with expected cut products (211 + 423 bp, see Table S1 for the primers used for the PCR and T7EI assay), and the efficiency reached a plateau after three rounds of selection. Sanger sequencing confirmed 70% *PARP1* mutagenesis generated in the selected PARP1m MDA‐MB‐231 cells (termed MDA‐MB‐231‐PARP1m; *n* = 20; Figure 1d). The large deletion was the only dominant mutation in the MDA‐MB‐231‐PARP1m cells, and it caused a frameshift at exon 7, thereby disrupting the PARP1 expression at the protein level (Figure S3). Also, since this TNBC cell line carries a triploid chromosome 1,^23^ our result indicated that two of the three chromosomes in the MDA‐MB‐231‐PARP1m cell line were edited, and one remained WT.

After confirming that our CRISPR editing and cell selection strategies could be applied to generate PARP1m cell lines, we applied these techniques to the BRCA1m TNBC line, MDA‐MB‐436. We obtained 85% *PARP1* gene disruption in MDA‐MB‐436 (*n* = 20), which resulted in a similar large deletion at exon 7 of *PARP1* (termed MDA‐MB‐436‐PARP1m; Figure 1c,d). This genetic disruption was accompanied by a significant reduction in PARP1 protein expression (Figure S3). Notably, this disruption was similar to that reported in a previous study.^24^ Nonetheless, after the introduction of CRISPR editing for these two rounds of enrichment, the edited MDA‐MB‐436 cells became unstable and formed heterogeneous populations. Since no HR template was introduced during the transfection, as expected, NHEJ was the likely pathway of DNA repair and caused *PARP1* mutagenesis.

To assess CRISPR/Cas9 off‐target effects, three primers were designed to match the most likely off‐target candidates with Cas‐OFFinder.^25^ A T7EI assay revealed that Cas9 did not induce any gene disruptions at these likely off‐target loci (Figure S4a). In addition to the Cas‐OFFinder prediction, we used another machine learning‐based algorithm, DeepCRISPR, to find the potential off‐targeting sites of our gRNA.^26^ According to the DeepCRISPR results, the gRNA that we designed had a relatively low possibility of introducing undesired gene editing (Figure S4b), yet we still chose the top four potential off‐target sites for further validation. Those sites were verified by amplicon‐based next generation sequencing. After removing the low‐quality reads, sequence variations at each site were detected with CRISPResso2.^27^ Again, the editing at those potential off‐targeting sites was minimal (modification rate < 0.5% for both MDA‐MB‐231‐PARP1m and MDA‐MB‐436‐PARP1m; Figure S4c). These results indicated the specificity of this system for *PARP1* targeting.

Using the two PARP1m TNBC lines with different *BRCA1* genetic profiles (WT and BRCA1m) and their *PARP1* WT counterparts as a basis of comparison, a luminescence‐based 2D cell viability assay was carried out. Three chemotherapeutic drugs approved for TNBC therapy were chosen: doxorubicin (DOX), gemcitabine (GEM), and docetaxel (DTX).^28^ These three drugs induce cell death through different mechanisms: DNA intercalation (DOX), DNA synthesis inhibition (GEM), and microtubular depolymerization (DTX).^29^ At 72 hr post‐treatment, the CRISPR/Cas9‐mediated *PARP1* mutagenesis significantly, and selectively, sensitized the MDA‐MB‐436‐PARP1m cells to chemotherapy (Figure 2). There was no synergism in the *BRCA1* WT conditions (MDA‐MB‐231 and MDA‐MB‐231PARP1m). All three drugs displayed similarly significant synergistic effects, despite potential differences in underlying mechanisms of action. Notably, the IC50 dose of GEM decreased most significantly between the MDA‐MB‐436 and MDA‐MB‐436‐PARP1m cells compared with the reduction of the IC50 doses for the other two drugs (Figure S5). Another study similarly reported that triple negative breast cancer cells were significantly sensitized to cell killing when gemcitabine was introduced in combination with a PARP1 inhibitor drug.^30^ At high drug concentrations, it is likely that too many of the cells were dead, making any synergy undiscernible. In contrast, at low drug concentrations, it is possible that the assay was not sufficiently sensitive to ascertain differences in cell viability. As such, the therapeutic window of synergy observed was likely restricted.

**Figure 2:**
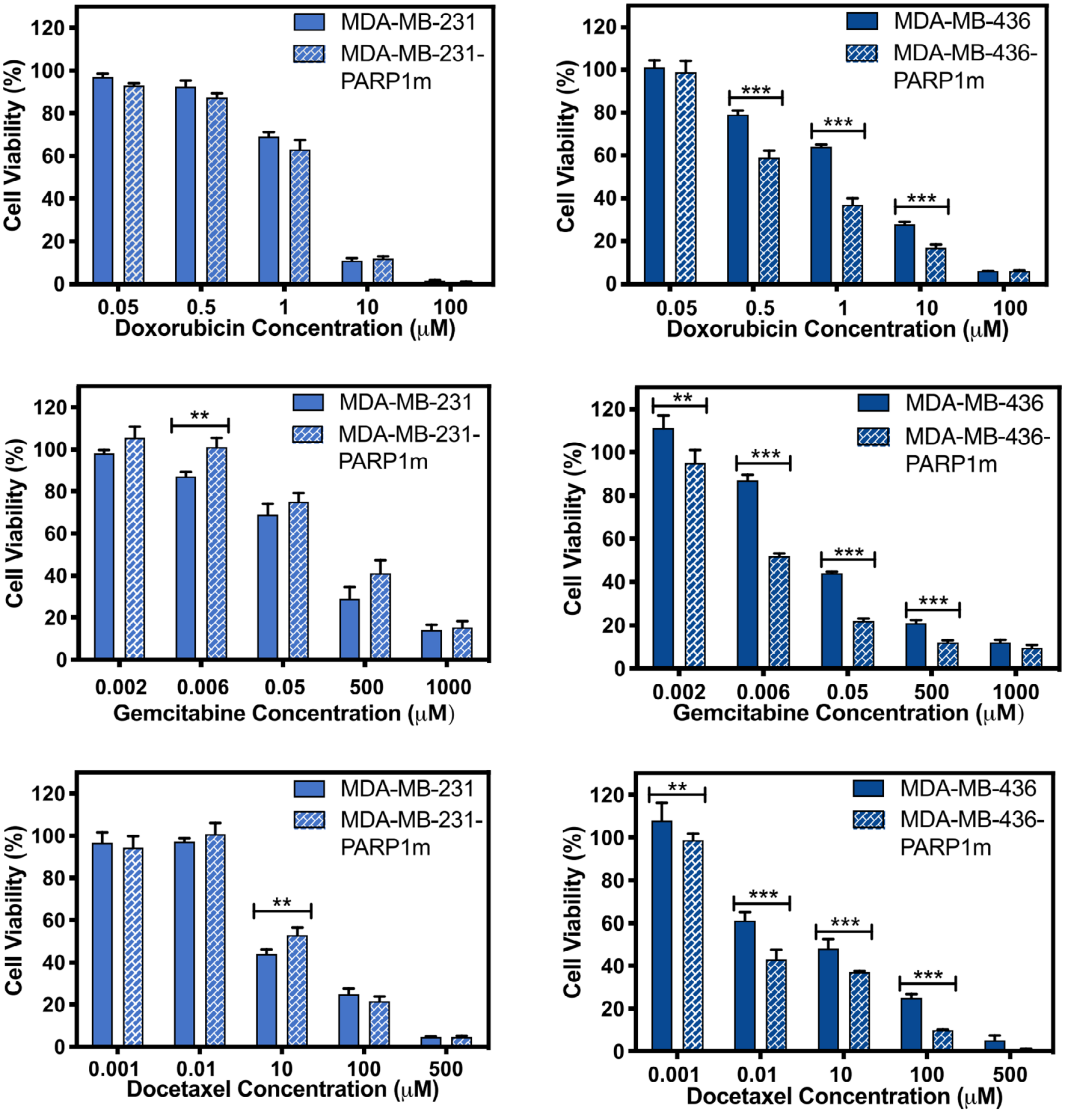
2D cell viability validation of *PARP1* disruption and chemotherapeutic drugs. Cell viability of *PARP1* WT (MDA‐MB‐231 and MDA‐MB‐436) and PARP1m (MDA‐MB‐436‐PARP1m and MDA‐MB‐436‐PARP1m) TNBC cells treated with (a) DOX, (b) GEM, and (c) DTX. Data are presented as mean ± standard deviation (SD). Significance was determined using *t*‐tests and presented as ** *p* < .01 and *** *p* < .001

It could be argued that the resultant cell death was not a result of the addition of chemotherapy but rather a result of the difference in cellular proliferation rates on account of the underlying *PARP1* mutagenesis itself. To explore this possible explanation, the cell proliferation of MDA‐MB‐436 and MDA‐MB‐436‐PARP1m cells was measured over the course of 72 hr. However, similar to the effect of PARP1 inhibitors on cell viability (Figure S1), *PARP1* disruption only slightly affected the overall doubling time of MDA‐MB‐436, although the difference was more pronounced at certain time points (Figure S6). This finding may indicate that the difference in cell death was not the sole result of the *PARP1* gene mutation.

Since the in vitro and clinical trial PARP1 inhibitor results conflict, an intermediate modality may help bridge the gap between the two approaches and shed light on the discrepancies. Tumor‐on‐a‐chip microfluidic models have emerged as a prominent technology to mimic in vivo physiological conditions with the fine‐tune in vitro control of the tumor microenvironment.^20^, ^31^ Although there is debate on whether 3D tumor‐on‐a‐chip models can faithfully represent the real tumor microenvironment and ultimately replace animal models, the platform facilitates a more systematic way to study each potential variable component (e.g., extracellular matrix, tumor‐stromal interaction, flow and hypoxia) that may affect the drug responses.^32^ In addition, the tumor‐on‐a‐chip platform enables screening in a high‐throughput manner with reduced sample volume, which may boost the drug screening process and reduce the cost for development.^31^

Therefore, a microfluidic model, consisting of the tumor microvasculature with human endothelial cells (Figures 3a,b),^15^ was used to validate the combinational synergy of PARP1m and chemotherapeutic drugs in a 3D setting. The TNBC cells, either MDA‐MB‐436 or MDA‐MB‐436‐PARP1m, were mixed with Matrigel® and seeded in each unit of the bottom chamber (*n* = 4; Figure 3c). After gelation, the human microvascular endothelial cells (HMVEC) were subsequently seeded in a confluent manner (Figure 3d). The cells were maintained in the device for 72 hr under 5% CO_2_ atmosphere at 37°C and supported with a continuous medium flow that was similar to the microvascular flow condition (100 μm/s). To visualize the cell under apoptosis, green fluorescent dye‐labeled caspase‐3 substrate was used, and the green fluorescence signal in each channel was recorded for 72 hr. The relative caspase‐3 activity was determined by normalizing the signal at each time point to the starting time, T_0_. When seeded in the device, there was no discernable difference in morphology between the MDA‐MB‐436 and MDA‐MB‐436‐PARP1m cells.

**Figure 3:**
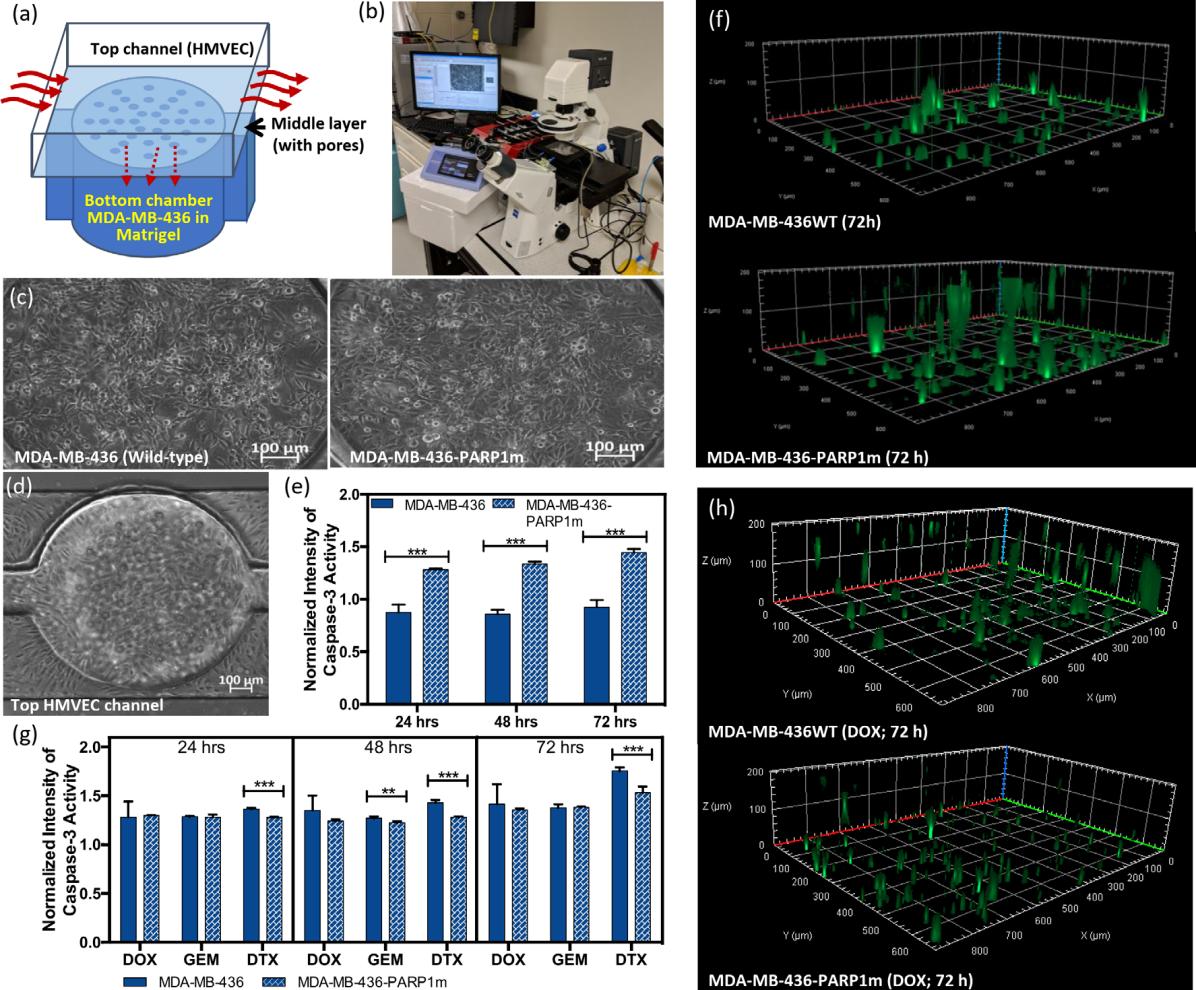
3D tumor‐on‐a‐chip system cell viability validation of *PARP1* disruption and chemotherapeutic drugs. (a) Schematic illustration of one unit in the presented tumor‐on‐a‐chip system. (b) Setup of the system integrated with pumps and microscope for in situ monitoring. (c) Representative images of MDA‐MB‐436 and MDA‐MB‐436‐PARP1m cells in the unit of the device. (d) The representative image of the top HMVEC layer. (e) The apoptotic pattern of MDA‐MB‐436 and MDA‐MB‐436‐PARP1m cells measured in the 3D system. (f) Representative images showing the apoptotic signals of MDA‐MB‐436 and MDA‐MB‐436‐PARP1m cells. (g) activity of MDA‐MB‐436 WT and MDA‐MB‐436‐PARP1m cells treated with DOX, GEM, and DTX. (h) Representative 3D‐reconstructed images of the unit treated with DOX. Green fluorescence represents the apoptotic cancer cells in the unit. Data are presented as mean ± SD. Significance was determined using *t*‐tests and presented as **p < .01 and ***p < .001

Notably, when cultured in the tumor‐on‐a‐chip system, a significant number of MDA‐MB‐436‐PARP1m underwent apoptosis without the addition of chemotherapeutic drugs, showing a similar fate of PARP1 inhibitor monotherapy found in preclinical and clinical validations (Figure 3e). In a 2017 phase III clinical trial consisting of 300 women (NCT02000622), for example, olaparib monotherapy induced toxicity, successfully halting the progression of BRCA1m breast cancer.^33^ Figure 3f shows a visual representation of the green fluorescent apoptotic cancer cells in each unit of the device seeded with either MDA‐MB‐436 and MDA‐MB‐436‐PARP1m cells, in which there are significantly more apoptotic cells in the PARP1m condition. The cells were treated with the IC50 doses of the three chemotherapeutic drugs determined in the 2D viability assay, yet, different from what we observed in the 2D system, there was no sensitization found in each combination in our 3D tumor‐on‐a‐chip system (Figure 3g,h). The three drug doses optimized for the 2D study could not be directly extrapolated to the tumor‐on‐a‐chip model, highlighting the challenge of dosing for drug screening.

Compared with the conventional 2D screening format, the 3D tumor‐on‐a‐chip platform provides a more clinically relevant microenvironment because the drug transport may be affected by both the endothelium of blood vessels and the limited diffusion in the extracellular matrix. In our previous study, we showed that both the extracellular matrix and flow played important roles in determining the drug response of cancer cells.^15^ These two variables, extracellular matrix and flow, were shown to be important in other studies as well^34^ and may explain why we observed a discrepancy in cell viability between the conventional 2D and our 3D tumor‐on‐a‐chip system.

Our 3D tumor‐on‐a‐chip results were consistent with the results from studies testing PARP1 inhibitors in combination with chemotherapy in breast cancer trials,^11^ and consistent with results from another in vitro study targeting ovarian cancer.^35^ Many TNBC studies have shown that the combinations do not provide benefit beyond the standard of care. Based upon reported in vitro synergism, paclitaxel and olaparib were tested in metastatic TNBC (NCT00707707). The results showed only partial antitumor activity but enhanced overall toxicity, neutropenia, and myelosuppression in patients who received combinational therapy in comparison to those who received either paclitaxel or olaparib alone.^36^ In a Phase II trial (NCT01506609), a combination of carboplatin and paclitaxel was compared with a combination of carboplatin, paclitaxel and veliparib. There was no difference in the progression‐free survival for the BRCA1m metastatic breast cancer patients. Similarly, in a recent Phase III clinical trial (NCT02032277), veliparib did not improve the efficacy of platinum‐based chemotherapy in TNBC patients with *BRCA1/2* germline mutations.^37^ These results support our findings in the 3D tumor‐on‐a‐chip system, implying that this drug screening platform may be able to provide additional therapeutic validation prior to clinical trials, potentially expediting drug translation.

In summary, CRISPR/Cas9 was designed and optimized to disrupt *PARP1*, the synthetic lethal pair of *BRCA1*. While the 2D in vitro results showed that CRISPR/Cas9‐mediated PARP1m sensitized the TNBC cells with BRCA1m to chemotherapeutic drugs, there was a dichotomy between the 2D and 3D tumor‐on‐a‐chip results, mirroring inconsistencies found in recent clinical trials. Collectively, our approach combining CRISPR/Cas9‐mediated mutagenous and a 3D tumor‐on‐a‐chip system may represent a better modeling strategy for drug screening. However, more investigation is needed to understand the mechanisms underlying these differences and drug dosing paradigms. Then, we can overcome these crucial barriers and determine the best way to optimize PARP1m‐based therapy for treating BRCA1m TNBC.

## DECLARATION OF INTEREST

U.S. patent 10,144,945 was issued to CUNY in 2018 for the 3D microfluidic cell array to create the tumor‐on‐chip used in this study.

## Supporting information


**Table S1** gRNA and primer sequences used in this study.
**Figure S1**. Responses of MDA‐MB‐231 and MDA‐MB‐436 cells to PARP inhibitors.
**Figure S2**. Sequencing validation of the all‐in‐one gRNA1‐encoding Cas9‐T2A‐EGFP plasmid.
**Figure S3**. PARP1/α‐Tubulin protein expression levels in MDA‐MB‐231‐PARP1m and MDA‐MB‐436‐PARP1m cells.
**Figure S4**. Off‐targeting validation of the gRNA1 used in this study.
**Figure S5**. Chemotherapy response of MDA‐MB‐436 and MDA‐MB‐436‐PARP1m cells.
**Figure S6**. Growth of MDA‐MB‐436 with and without *PARP1* disruption.
