# Supplementary material for: CRISPR/Cas9‐mediated mutagenesis to validate the synergy between PARP1 inhibition and chemotherapy in BRCA1‐mutated breast cancer cells
Source: Bioeng Transl Med. 2020 Jan 2;5(1):e10152. doi: 10.1002/btm2.10152 (PMC6971465; doi:10.1002/btm2.10152)
Supplement: Supplementary file 1 — Table S1 gRNA and primer sequences used in this study. Figure S1. Responses of MDA‐MB‐231 and MDA‐MB‐436 cells to PARP inhibitors. Figure S2. Sequencing validation of the all‐in‐one gRNA1‐encoding Cas9‐T2A‐EGFP plasmid. Figure S3. PARP1/α‐Tubulin protein expression levels in MDA‐MB‐231‐PARP1m and MDA‐MB‐436‐PARP1m cells. Figure S4. Off‐targeting validation of the gRNA1 used in this study. Figure S5. Chemotherapy response of MDA‐MB‐436 and MDA‐MB‐436‐PARP1m cells. Figure S6. Growth of MDA‐MB‐436 with and without PARP1 disruption. [file BTM2-5-e10152-s001.pdf]

Supporting Information for

CRISPR/Cas9-Mediated Mutagenesis to Validate the Synergy between PARP1 Inhibition and Chemotherapy in *BRCA1*-Mutated Breast Cancer Cells

Rachel L. Mintz[1], Yeh-Hsing Lao[1], Chun-Wei Chi[2], Siyu He[1], Mingqiang Li[1,3], Chai Hoon Quek[1], Dan Shao[1], Boyuan Chen[1], Jing Han[1,4], Sihong Wang[2] and Kam W. Leong[1,5*]

[1] Department of Biomedical Engineering, Columbia University, New York, NY 10027, USA
[2] Department of Biomedical Engineering, CUNY- City College of New York, New York, NY 10031, USA
[3] Laboratory of Biomaterials and Translational Medicine, The Third Affiliated Hospital, Sun Yat-sen University, Guangzhou 510630, China
[4] State Key Laboratory of Microbial Resources, Institute of Microbiology, Chinese Academy of Sciences, Beijing 100101, China
[5] Department of Systems Biology, Columbia University Medical Center, New York, NY 10032, USA
* Corresponding author: Prof. Kam W. Leong (e-mail: kam.leong@columbia.edu)

This document includes

Experimental Section
Table S1. gRNA and primer sequences used in this study.
Figure S1. Responses of MDA-MB-231 and MDA-MB-436 cells to PARP inhibitors.
Figure S2. Sequencing validation of the all-in-one gRNA1-encoding Cas9-T2A-EGFP plasmid.
Figure S3. PARP1/α-Tubulin protein expression levels in MDA-MB-231-PARP1m and MDA-MB-436-PARP1m cells.
Figure S4. Off-targeting validation of the gRNA1 used in this study.
Figure S5. Chemotherapy response of MDA-MB-436 and MDA-MB-436-PARP1m cells.
Figure S6. Growth of MDA-MB-436 with and without *PARP1* disruption.

Experimental Section

CRISPR/Cas9 gRNA Design. The online, published tool CHOPCHOP was used for selecting gRNA candidates to target the *PARP1* gene,[1] with the default Cas9 targeting setting and a preference of *Streptococcus pyogenes* Cas9's NGG PAM motif and 5'-GG in gRNA's targeting sequence. The top three gRNA candidates with the fewest off-target effects, defined as up to two mismatches in the first 20 base pairs were chosen.[2] The gRNA1 with the greatest gene disruption efficiency was subsequently cloned into an all-in-one Cas9-T2A-EGFP vector (Addgene plasmid# 48138, Watertown, MA).[3]

Cell Culture. The MDA-MB-231 and MDA-MB-436 cells were purchased from American Type Culture Collection (Manassas, VA). These two cell lines and their derived cell lines, were maintained in the complete medium, composed of Dulbecco's Modified Eagle Medium (Thermo Fisher, Waltham, MA) with 10% fetal bovine serum (FBS; Atlanta Biologicals, Flowery Branch, GA), 100 U/mL of penicillin-streptomycin (Thermo Fisher) and 1× MEM Non-Essential Amino Acids (Thermo Fisher) at 37°C with an atmosphere of 5% CO_2.

Cell Selection and PARP1 Disruption Validation. The gRNA1-encoding Cas9-T2A-EGFP plasmid was transfected using Lipofectamine 3000 (Thermo Fisher), and the live GFP^+ cells were then sorted at 24 h post-transfection. The cells carrying *PARP1* mutation were selected using the low-density seeding approach. Multiple rounds of selection were carried out to enrich the populations with *PARP1* mutation. To validate the gene disruption, the genomic DNA was extracted, and the Cas9-targeting site was amplified by a 2-step PCR using Takara Terra hot-start polymerase (98°C for 2 min for enzyme activation; 30 cycles of 98°C for 10 s and 68°C for 1 min). After amplification, the PCR product was purified using Takara NucleoSpin Gel and PCR Clean-up kit (Japan), and the DNA concentration was determined by UV-VIS. The T7EI assay was then carried out by following our previously established protocol.[4] Briefly, 200 ng of the PCR product was first reannealed to generate heterogenous hybrids. The reannealed sample was subsequently incubated with the T7 endonuclease I (New England Biolabs, Ipswich, MA) at 37°C for 20 min. The PCR and T7EI products were run and visualized using a 2% ethidium bromide-prestained TAE-agarose gel.

To determine the disruption efficiency, the purified PCR product was cloned into a pUC19 vector (Takara) and transformed to the Clontech Stellar competent cells (Takara). After overnight culture of the transformed bacteria at 37°C, the clone was amplified using the same, aforementioned 2-step PCR method for 35 cycles, and the products were purified using the Exo-SAP IT enzymatic reaction (Thermo Fisher). Sanger sequencing was done by Eton Bioscience (Union, NJ).

Off-targeting validation using DeepCRISPR and amplicon NGS. The top 4 off-target sites predicted by DeepCRISPR[5] were amplified using the same Terra polymerase. After PCR purification, the concentration of each purified product was determined using the Quant-iT PicoGreen dsDNA Assay (Thermo Fisher). The products were sequenced by Genewiz (South Plainfield, NJ). The pair-end sequencing result and modification rate in each amplicon were analyzed with the CRISPResso2 pipeline.[6]

2D Cell Viability Assays. Cells were seeded in a white-colored, luminescent 96-well plate (n = 4) to achieve 70-80% confluency in each well. At 24 h post-seeding, the cells were treated with a chemotherapeutic drug (DOX, GEM or DTX) or a PARP inhibitor (olaparib or veliparib) for an additional 72 h. The cell viability was determined using CellTiter-Glo reagent (Promega, Madison, WI) by following the manufacturer's instructions.

3D Validation using the Tumor-on-a-chip System. The TNBC cells mixed with growth factor-reduced Matrigel (Corning, Corning, NY) were first seeded in each unit of the tumor-on-a-chip device[7] (n = 4). At 24 h post-seeding, HMVEC cells (Lonza, Switzerland) were seeded on the top layer. Afterwards, the cells were continuously treated with chemotherapeutic drugs (DOX, GEM or DTX) for 72 h. The apoptotic cells were visualized using a green caspase-3 substrate (Biotium, Fremont, CA), and the activity was measured and normalized to the control at T_0.

Table S1. gRNA and primer sequences used in this study.

Name	Sequence	Purpose	Expected Amplicon (bp)
gRNA1	GGTCCAAGATCTGCAGCCAG	Cas9 targeting	N/A
gRNA2	GGCAGAGCCTGTTGAAGTTG	Cas9 targeting	N/A
gRNA3	GGTAAGCACAGGGCTACCAG	Cas9 targeting	N/A
Cas9/gRNA1 On-Target Primers	Forward: GCAGGAACACTTGGAAATAAGG<br>Reverse: TTTGCAAGCAACATTTTACACC	PCR	634<br>T7EI: 211 + 423
Cas9/gRNA2 On-Target Primers	Forward: CCACTGTAGGTCTTCAGGAACC<br>Reverse: TTGTTCTCATTCCCATCATCTG	PCR	766<br>T7EI: 273 + 493
Cas9/gRNA3 On-Target Primers	Forward: AGAGGAGACATGGTTGGCTAAG<br>Reverse: AGAGGGTAACCAACGTCTTCAA	PCR	602<br>T7EI: 257 + 345
Cas9/gRNA1 Off-Target Primer 1	Forward: CGGTACCCGGGGATCCAGACTGAGGTCGCCTTGC<br>Reverse: CGACTCTAGAGGATCCACTGAGCTGACCAAGATGATTAC	PCR/Sequencing	PCR: 622<br>T7E1: 117 + 505
Cas9/gRNA1 Off-Target Primer 2	Forward: CGGTACCCGGGGATCGCTGAGACATGTTAGGGAAAGC<br>Reverse: CGACTCTAGAGGATCTATGGGGCTTGGCATTGTTGATA	PCR/Sequencing	PCR: 922<br>T7E1: 143 + 779
Cas9/gRNA1 Off-Target Primer 3	Forward: CGGTACCCGGGGATCCCCGCGGATTATGTCCCATT<br>Reverse: CGACTCTAGAGGATCGGGGGTGTTTTGGTTCTAGC	PCR/Sequencing	PCR: 617<br>T7EI: 143 + 474
Cas9/gRNA1 DeepCRISPR OT1 primers	Forward: TGTTCACGGGTCAGACGAG<br>Reverse: TTGATTTGTGGACATGGACCCT	PCR/NGS	200
Cas9/gRNA1 DeepCRISPR OT2 primers	Forward: CCCTTCGCTCGGATGAGG<br>Reverse: TTGCCCCTTAGATTGAGTATCCTG	PCR/NGS	200
Cas9/gRNA1 DeepCRISPR OT3 primers	Forward: CTTGCAAGGAATTCAAAGGCTCT<br>Reverse: CTCCTGTGCAGTCAGAACAAC	PCR/NGS	200
Cas9/gRNA1 DeepCRISPR OT4 primers	Forward: GATGCAGAAAGAGCCAAAAACCA<br>Reverse: CGAAAGACCCAGAACCCCTG	PCR/NGS	200
Cas9/gRNA1 Sequencing Primers	Forward: CGGTACCCGGGGATCGCAGGAACACTTGGAAATAAGG<br>Reverse: CGACTCTAGAGGATCTTTGCAAGCAACATTTTACACC	Sequencing	664

*The adaptor sequence used for cloning for Sanger sequencing is underlined.

Supplementary Figures

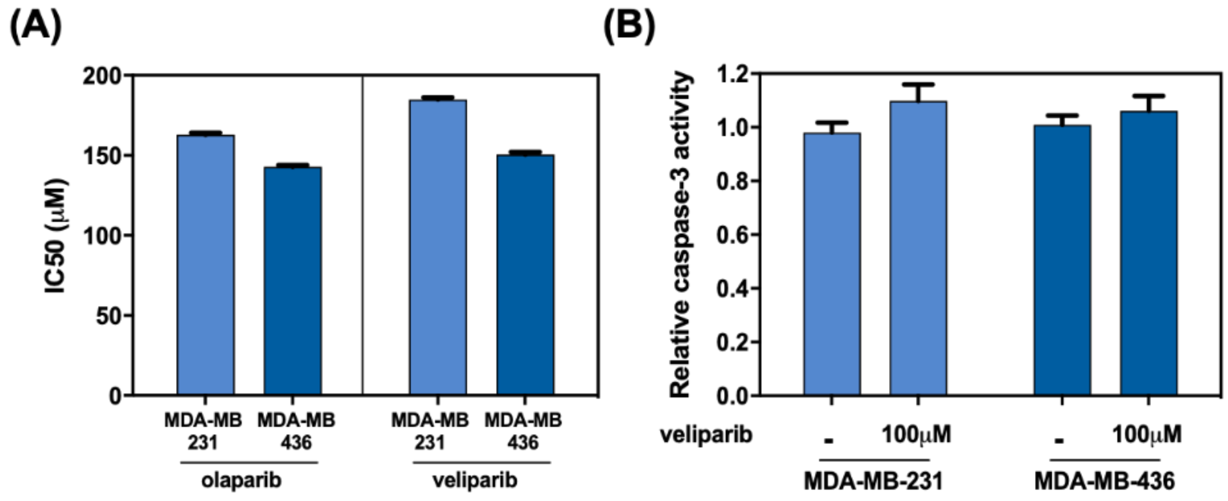

Figure S1. Responses of MDA-MB-231 and MDA-MB-436 cells to PARP inhibitors. (A) IC50 doses of olaparib and veliparib in the 2D *in vitro* system. Data represented as average ± SD (*n* = 4). (B) Response of MDA-MB-231 and MDA-MB-436 when treated with veliparib on the 3D system (w/o EC barrier). Data represented as average ± SD (*n* = 8).

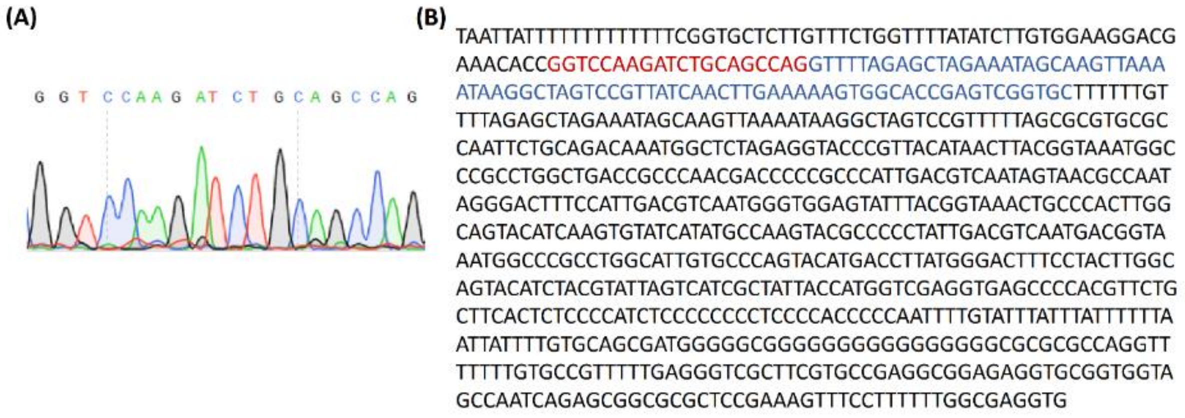

Figure S2. Sequencing validation of the all-in-one gRNA1-encoding Cas9-T2A-EGFP plasmid. (A) Fluorescent spectrum of the sequenced result of gRNA1's 20mer targeting region. (B) Sanger sequencing result (using LKO1 5" primer to target human U6 promoter of the plasmid). The 20mer targeting region and the gRNA backbone are highlighted in red and blue, respectively.

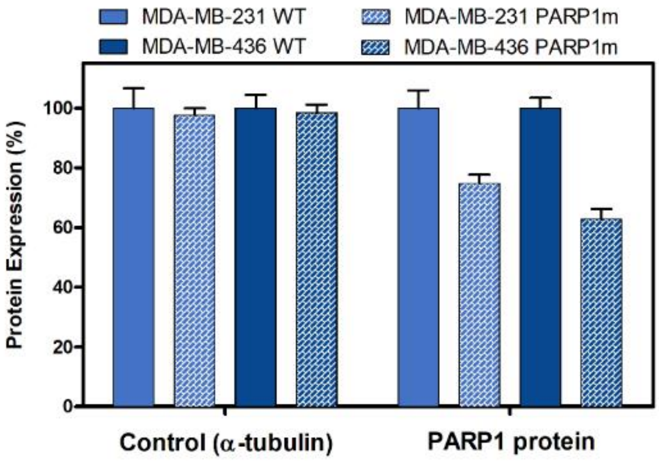

Figure S3. PARP1/α-Tubulin protein expression levels in MDA-MB-231-PARP1m and MDA-MB-436-PARP1m cells. The protein expression was normalized to the wild-type controls (MDA-MB-231WT or MDA-MB-436WT). Data are presented as average ± SD (n = 4).

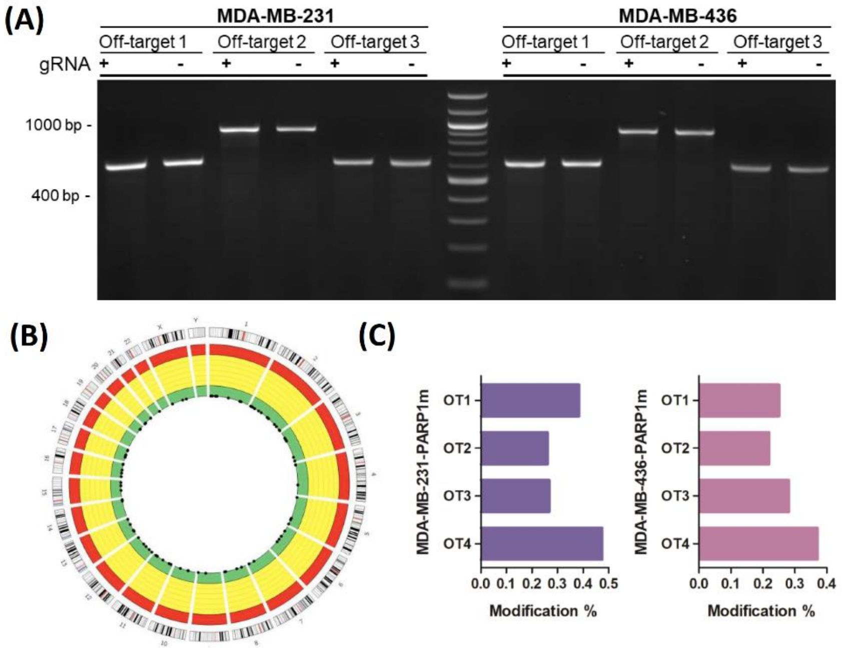

Figure S4. Off-targeting validation of the gRNA1 used in this study. (A) OFF-targeting validation using the T7EI assay on the top 3 potential off-target sites predicted by CasOFFinder. (B) Potential off-target sites predicted by DeepCRISPR. (C) Off-targeting validation using amplicon NGS on the top 4 off-target sites predicted by DeepCRISPR. The modification rate at each site was analyzed by the CRISPResso2 pipeline.

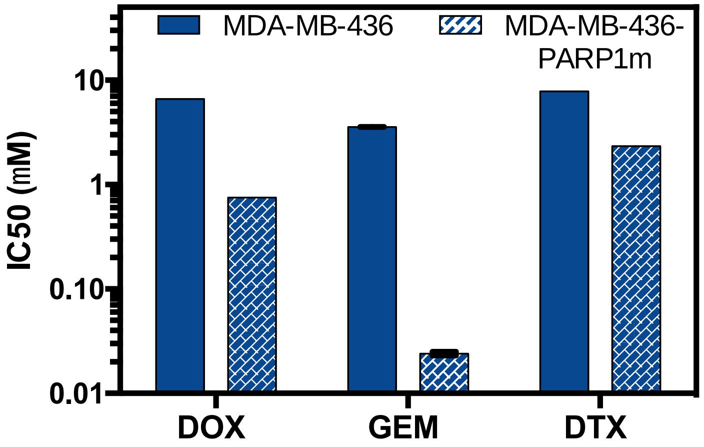

Figure S5. Chemotherapy response of MDA-MB-436 and MDA-MB-436-PARP1m cells. Data are presented as average ± SD (*n* = 4).

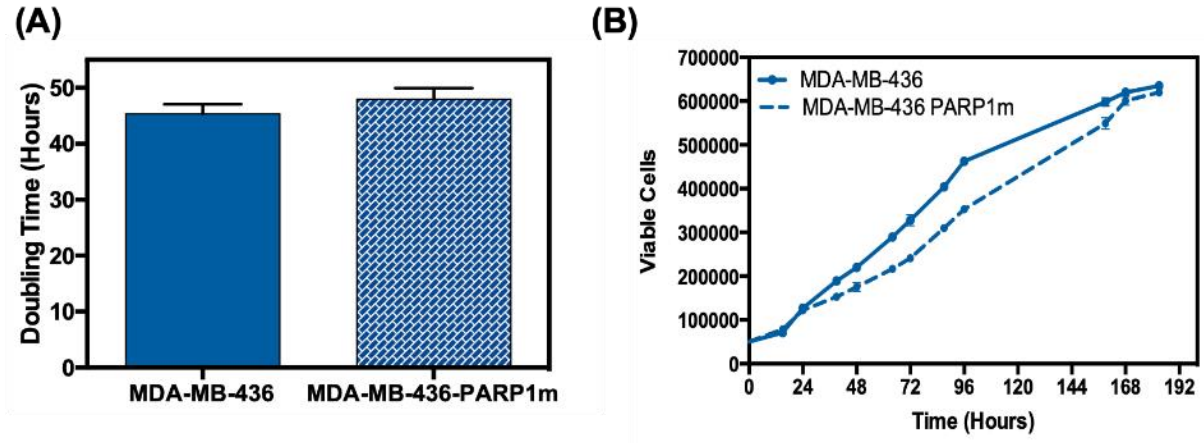

Figure S6. Growth of MDA-MB-436 cells with and without *PARP1* disruption. **(A)** Doubling time of MDA-MB-436 and MDA-MB-436-PARP1m cells (B) Growth curves of MDA-MB-436 and MDA-MB-436-PARP1m cells. Data are presented as average ± SD (*n* = 3).

References

1. **1.** Montague TG, Cruz JM, Gagnon JA, Church GM, Valen E, CHOPCHOP: a CRISPR/Cas9 and TALEN web tool for genome editing. *Nucleic Acids Res.* 2014;42:W401-W407. https://doi.org/10.1093/nar/gku410.
2. **2.** Hsu PD, Scott DA, Weinstein JA, Ran FA, Konermann S, Agarwala V, Li Y, Fine EJ, Wu X, Shalem O, Cradick TJ, Marraffini LA, Bao G, Zhang F, DNA targeting specificity of RNA-guided Cas9 nucleases. *Nat. Biotechnol.* 2013;31(9):827-832. https://doi.org/10.1038/nbt.2647.
3. **3.** Ran FA, Hsu PD, Wright J, Agarwala V, Scott DA, Zhang F, Genome engineering using the CRISPR-Cas9 system. *Nat. Protoc.* 2013;8(11):2281-2308. https://doi.org/10.1038/nprot.2013.143.
4. **4.** Lao Y-H, Li MQ, Gao MA, Shao D, Chi C-W, Huang DT, Chakraborty S, Ho TC, Jiang WQ, Wang HX, Wang SH, Leong KW, HPV Oncogene manipulation using nonvirally delivered CRISPR/Cas9 or *Natronobacterium gregoryi* Argonaute. *Adv. Sci.* 2018;5(7):1700540. https://doi.org/10.1002/advs.201700540.
5. **5.** Chuai GH, Ma HH, Yan JF, Chen M, Hong NF, Xue DY, Zhou C, Zhu CY, Chen K, Duan B, Gu F, Qu S, Huang DS, Wei J, Liu Q, DeepCRISPR: optimized CRISPR guide RNA design by deep learning. *Genome Biol.* 2018;19:80. https://doi.org/10.1186/s13059-018-1459-4.
6. **6.** Clement K, Rees H, Canver MC, Gehrke JM, Farouni R, Hsu JY, Cole MA, Liu DR, Joung JK, Bauer DE, Pinello L, CRISPResso2 provides accurate and rapid genome editing sequence analysis. *Nat. Biotechnol.* 2019;37(3):224-226. https://doi.org/10.1038/s41587-019-0032-3.
7. **7.** Dereli-Korkut Z, Akaydin HD, Ahmed AHR, Jiang XJ, Wang SH, Three dimensional microfluidic cell arrays for *ex vivo* drug screening with mimicked vascular flow. *Anal. Chem.* 2014;86(6):2997-3004. https://doi.org/10.1021/ac403899j.
